# Targeting the Early Endosome-to-Golgi Transport of Shiga Toxins as a Therapeutic Strategy

**DOI:** 10.3390/toxins12050342

**Published:** 2020-05-22

**Authors:** Danyang Li, Andrey Selyunin, Somshuvra Mukhopadhyay

**Affiliations:** Division of Pharmacology & Toxicology, College of Pharmacy, Institute for Cellular & Molecular Biology, and Institute for Neuroscience, The University of Texas at Austin, Austin, TX 78712, USA; danyangli@utexas.edu (D.L.); andrey.selyunin@austin.utexas.edu (A.S.)

**Keywords:** early endosome-to-Golgi trafficking, late endosomes, lysosomes, HOPS (homotypic fusion and vacuole protein sorting), autophagy, small-molecule inhibitor, Golgi phosphoprotein 4 (GPP130), tamoxifen, manganese

## Abstract

Shiga toxin (STx) produced by *Shigella* and closely related Shiga toxin 1 and 2 (STx1 and STx2) synthesized by Shiga toxin-producing *Escherichia coli* (STEC) are bacterial AB_5_ toxins. All three toxins target kidney cells and may cause life-threatening renal disease. While *Shigella* infections can be treated with antibiotics, resistance is increasing. Moreover, antibiotic therapy is contraindicated for STEC, and there are no definitive treatments for STEC-induced disease. To exert cellular toxicity, STx, STx1, and STx2 must undergo retrograde trafficking to reach their cytosolic target, ribosomes. Direct transport from early endosomes to the Golgi apparatus is an essential step that allows the toxins to bypass degradative late endosomes and lysosomes. The essentiality of this transport step also makes it an ideal target for the development of small-molecule inhibitors of toxin trafficking as potential therapeutics. Here, we review the recent advances in understanding the molecular mechanisms of the early endosome-to-Golgi transport of STx, STx1, and STx2, as well as the development of small-molecule inhibitors of toxin trafficking that act at the endosome/Golgi interface.

## 1. Introduction

Infections caused by *Shigella* bacteria that produce Shiga toxin (STx) and Shiga toxin-producing *Escherichia coli* that produce Shiga toxin 1 and 2 (STx1 and STx2) are major causes of water- and food-borne disease in the world [1,2]. *Shigella* infections predominate in developing countries due to poor sanitation conditions [2]. In contrast, STEC infections are more commonly seen in North America, Europe, and Australia [3]. Infected patients initially develop gastrointestinal disease [1,4]. In a subset, the released toxins enter the bloodstream and target renal microvascular endothelial and tubular epithelial cells to cause life-threatening hemolytic uremic syndrome [1,4,5]. While *Shigella* infections can be treated with antibiotics, drug resistance is an emerging problem [2]. In the case of STEC-induced disease, there are no definitive treatments, and antibiotic therapy is contraindicated because it may increase toxin release from the bacteria and enhance the risk of severe renal damage [1,6,7]. Overall, there is an urgent need to develop effective interventions to counter disease caused by these bacterial toxins.

STx, STx1, and STx2 belong to the AB_5_ class of bacterial exotoxins and are formed by the association of an A-subunit with a pentameric B-subunit [8,9]. The A-subunit has ribosomal RNA *N*-glycosidase activity and induces toxicity by inhibiting protein synthesis in host cells [4,8,9]. The pentameric B-subunits mediate retrograde trafficking from the cell surface to the cytosol [4,8,9,10]. STx1 is essentially identical to STx with 100% conservation in the B subunit and a conservative serine-to-threonine substitution in the A subunit [9,11]. However, STx2 shares only ~55% sequence identity with STx and STx1 [9,11]. Notably, with STEC infections, STx2 is the more disease-relevant toxin; the Mater. Today Proc. median lethal dose (LD_50_) of STx2 is ~400-fold lower than that of STx1, and production of STx2 is associated with disease severity [1,12,13].

**Figure 1:**
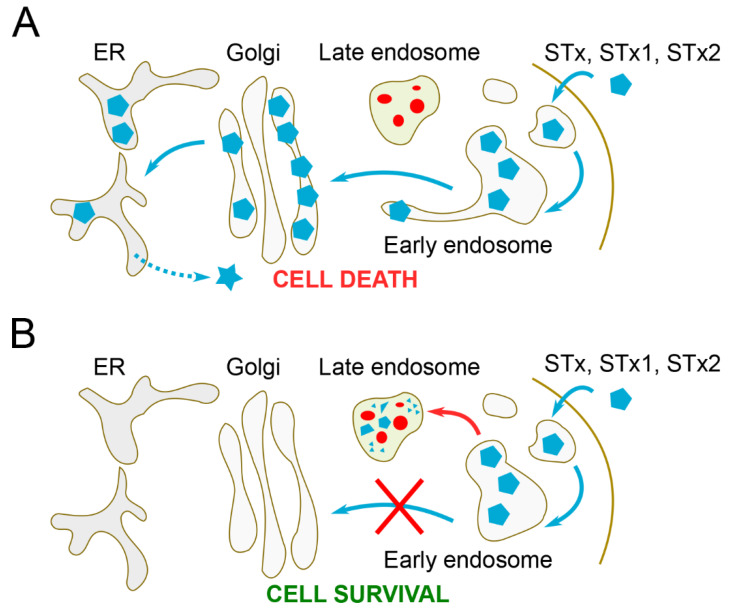
Retrograde transport of Shiga toxin (STx), STx1, and STx2. (**A**) Direct early endosome-to-Golgi transport allows the toxins to evade degradation in late endosomes. (**B**) When early endosome-to-Golgi transport is inhibited, the toxins are routed to, and degraded in, late endosomes. ER, endoplasmic reticulum.

STx, STx1, and STx2 traffic via an elaborate retrograde route to reach the cytosol of host cells. The steps involved are, sequentially, binding to globotriaosylceramide, the cell-surface glycosphingolipid receptor for the toxins, followed by endocytosis and internalization to early endosomes, direct transit from early endosomes to the Golgi apparatus, and delivery to the endoplasmic reticulum from where the A-subunits are translocated to the cytosol (Figure 1A) [9,14,15,16]. The early endosome-to-Golgi transport step is critical as it allows the toxins to bypass degradative late endosomes and lysosomes (Figure 1A) [9,14]. As retrograde trafficking is essential for toxicity, there is a high interest in developing inhibitors of toxin transport for therapy [9,17,18,19,20]. Targeting the early endosome-to-Golgi step has particular appeal, as inhibition of toxin transport at this step is expected to reroute the toxins to degradative late endosomes/lysosomes (Figure 1B). Here, we review current understanding of the molecular mechanisms of the early endosome-to-Golgi trafficking of STx, STx1, and STx2, and we summarize how basic science advances are being utilized for the development of small-molecule inhibitors of toxin transport.

## 2. Early Endosome-to-Golgi Transport of STx and STx1

Although STx2 is more toxic and disease-relevant, most studies over the last three decades focused on the retrograde trafficking of STx/STx1 B-subunit (STxB/STx1B) (the B subunits of STx and STx1 are identical) [9,11,21,22]. Indeed, STxB/STx1B was one of the first proteins shown to undergo direct early endosome-to-Golgi transport [23]. There is a fairly sophisticated understanding of the trafficking mechanisms of STxB/STx1B at the early endosome/Golgi interface, and STxB/STx1B was developed as a morphological and quantitative biochemical tool to study retrograde trafficking itself. Factors required for the early endosome-to-Golgi trafficking of STxB/STx1B are provided in Table 1 and described below.

### 2.1. GPP130—The Endosomal Receptor for STxB/STx1B

**Figure 2:**
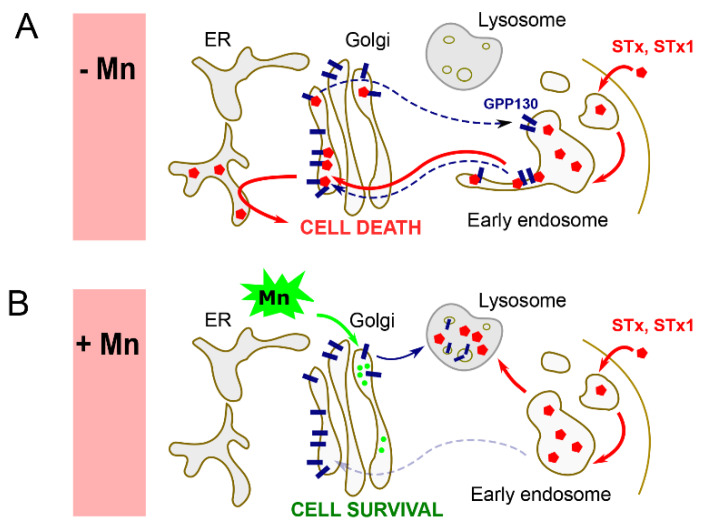
Golgi phosphoprotein 4 (GPP130) is the endosomal receptor for STx/STx1. (**A**) Under control conditions, STxB/STx1B binds the luminal domain of GPP130, which allows the toxin to traffic to the Golgi from early endosomes. (**B**) Manganese treatment induces lysosomal degradation of GPP130. Under these conditions, STxB/STx1B fails to exit early endosomes and is also degraded in lysosomes. Mn, manganese; ER, endoplasmic reticulum.

STxB/STx1B are soluble proteins that are contained within the endosomal lumen and cannot directly access cytosolic trafficking factors that mediate early endosome-to-Golgi trafficking. Instead, the toxins rely on the host protein Golgi phosphoprotein 4 (GPP130), which serves as the endosomal sorting receptor for STxB/STx1B (Figure 2A) [10,19]. GPP130 is a single-pass transmembrane protein that constitutively cycles between the *cis*-Golgi and early endosomes (Figure 2A) [24,25,26]. STxB/STx1B directly binds the luminal domain of GPP130 with a K_d_ of 150 nM [19]. Depletion of GPP130 inhibits the early endosome-to-Golgi transport of STxB/STx1B (Figure 2B) [19]. This effect is rescued by expression of full-length GPP130, but not by GPP130 mutants that lack the binding site for STxB/STx1B or that cannot cycle between the Golgi and early endosomes [19]. Furthermore, the cytosolic domain of GPP130 interacts with the SNARE syntaxin 5 [27]. Syntaxin 5 is required for the early endosome-to-Golgi transport of STxB/STx1B (see below), and the GPP130–syntaxin 5 interaction is critical to support STxB/STx1B transport [27]. Overall, binding of STxB/STx1B to its endosomal receptor, binding of the endosomal receptor to a critical cytosolic trafficking factor, and cycling of the receptor itself are necessary for toxin transport (Figure 2A). GPP130 is the first, and to date only, endosomal receptor identified for an AB_5_ toxin.

### 2.2. Cholesterol and Membrane Microdomains

Cholesterol also plays an important role in the retrograde transport of STxB/STx1B (Table 1). In HeLa cells, STxB/STx1B is associated with cholesterol-rich membrane microdomains, and disruption of these microdomains by cholesterol extraction strongly inhibits the early endosome-to-Golgi transport of STxB/STx1B [28]. Interestingly, it is not yet clear whether STxB/STx1B remains bound to globotriaosylceramide, the cell-surface receptor for STxB/STx1B [16], in early endosomes and the Golgi, but globotriaosylceramide is also enriched in cholesterol-rich microdomains [29] and could contribute to the retention of STxB/STx1B in these regions (note that globotriaosylceramide is the cell-surface receptor while GPP130 is the endosomal receptor for STxB/STx1B).

### 2.3. Clathrin and Retromer Coats and Accessory Proteins

Clathrin has a well-established role in retrograde transport from early endosomes to the Golgi. STxB/STx1B was observed in clathrin-containing endosomal tubules [30], and antisense RNA against clathrin inhibits STxB/STx1B from exiting early endosomes [30,31]. The clathrin adaptor epsinR is also found in STxB/STx1B-containing early endosomal tubules [30]. EpsinR is proposed to serve as a multivalent linker between early endosome membrane lipids and clathrin coats. Indeed, transport of STxB/STx1B from early endosomes to the Golgi is inhibited in epsinR small interfering RNA (siRNA)-treated cells [30], and overexpression of wild-type epsinR impairs STxB/STx1B transport [30].

Additional factors that regulate adaptor recruitment also contribute to the retrograde transport of STxB/STx1B. One example is OCRL1, a phosphatidylinositol 4,5-bisphosphate 5-phosphatase that is associated with clathrin-coated, Golgi-directed intermediates [32,52]. OCRL1 catalyzes the production of phosphatidylinositol 4-phosphate on early endosomal membranes [32], thus modulating adaptor recruitment. Additionally, OCRL1 directly interacts with clathrin and promotes clathrin assembly in vitro [32]. Overexpression of OCRL1 blocks early endosome-to-Golgi transport of STxB/STx1B [32].

Retromer is a heteropentameric coat complex composed of a sorting nexin (SNX) dimer and a Vps26–Vps29–Vps35 heterotrimer, which is also known as the cargo recognition complex [53]. SNX proteins have PX domains that interact with endosomal phosphatidylinositol 3-phosphate and phosphatidylinositol 3,5-bisphosphate, and BAR domains that sense and/or generate membrane curvature [53]. siRNA-mediated silencing of SNX1 or SNX2 leads to a significant reduction in the early endosome-to-Golgi transport of STxB/STx1B [33,34]. Furthermore, retrograde transport of STxB/STx1B is also impaired in Vps26 siRNA-treated cells [35]. Thus, retromer plays a critical role in STxB/STx1B transport. EHD3 and its early endosomal interaction partner rabenosyn-5 regulate recruitment of retromer to early endosomes and are also required for STxB/STx1B transport [36].

Clathrin and retromer coats are believed to function in sequential transport steps, rather than in parallel, since disruption of either causes a nearly complete block in retrograde transport of STxB/STx1B [30,35]. In clathrin-depleted cells, STxB/STx1B colocalizes with transferrin receptor, a canonical marker for early endosomes, while, in Vps26-depleted cells, STxB/STx1B is found in transferrin receptor-free endosomal tubules [30,35]. Based on these observations, a two-step model for endosomal retrograde sorting is proposed, with clathrin mediating the initial membrane deformation and retromer sequentially stabilizing and processing the nascent tubules [35]. The same group later identified molecular articulation between clathrin and retromer [37]. Clathrin interactor Hrs was shown to interact with SNX1, which may lead to the recruitment of retromer to the pre-deformed early endosome membrane, while the SNX1 interactor RME-8 was shown to recruit the clathrin-uncoating ATPase Hsc-70, which may facilitate substitution of clathrin by retromer [37].

### 2.4. GTPase Dynamin

The GTPase dynamin contributes to the retrograde transport of STxB/STx1B. The early endosome-to-Golgi transport of STxB/STx1B is inhibited by overexpression of a dominant-negative dynamin mutant that is unable to bind and hydrolyze GTP [31].

### 2.5. Cytoskeletal Factors

The early endosome-to-Golgi transport of STxB/STx1B also requires the actin and microtubule cytoskeleton, the dynein motor, the Rho GTPase Cdc42, and the Cdc42 GTPase-activating protein (GAP) ARHGAP21 [38,39]. Indeed, transport of STxB/STx1B from the endosomal compartment to the Golgi is blocked by acute nocodazole treatment that disrupts microtubules, cytochalasin D treatment that disrupts the actin cytoskeleton, expression of constitutively active or dominant-negative mutants of Cdc42, siRNA-mediated knockdown of Cdc42, or short hairpin RNA (shRNA)-mediated knockdown of ARHGAP21 [38,39].

### 2.6. Tethering, Docking, and Fusion with the Golgi

Rab family GTPases together with their regulators define the structural and functional identity of intracellular organelles [54]. Rab11 and Rab6a’ are implicated in the endosome-to-Golgi transport of STxB/STx1B [40,41,42]. Antibody against Rab11 or overexpression of wild-type, GTPase-deficient, or dominant-negative Rab11 inhibits the delivery of STxB/STx1B from endosomes to the Golgi [40,41]. Antibody or siRNA-mediated inhibition of Rab6a’ or overexpression of dominant-negative Rab6a’ also leads to the accumulation of STxB/STx1B in the endosomal compartment [41,42]. Additionally, a series of Rab GTPase-activating proteins (EVI5, RN-tre, TBC1D10A–C, and TBC1D17) are reported to play a role in the transport of STxB/STx1B to the Golgi, but whether these proteins, and their associated Rabs, are *bona fide* regulators of toxin transport at the early endosome-to-Golgi transport step is unclear [55].

Golgins are long coiled-coil proteins that are anchored to the Golgi membrane through the C terminus [56]. These highly flexible proteins are not only responsible for linking Golgi cisternae and ministacks but also tethering vesicles and Rab effectors [56]. Early endosome-to-Golgi transport of STxB/STx1B requires at least four golgins: golgin-97, golgin-245, GCC185, and TMF [43,44,45,46]. All four localize to the *trans*-Golgi network, with golgin-97, golgin-245, and GCC185 recruited by Arl1 and TMF by Rab6 [56]. RNA interference of Arl1 or any of these golgins causes defects in the retrograde transport of STxB/STx1B from early endosomes to the Golgi [43,44,45,46]. The GARP complex is another tethering factor required for the transport of STxB/STx1B to the Golgi. In cells depleted of GARP by siRNA, STxB/STx1B fails to traffic to the Golgi and accumulates in a population of small endosome-like structures [47].

SNARE proteins are transmembrane proteins that mediate fusion of vesicles with target membranes [57]. The early endosomes-to-Golgi transport of STxB/STx1B relies on two SNARE complexes [22]. One is composed of syntaxin 5, Ykt6, GS15, and GS28, and the other of syntaxin 6, syntaxin 16, Vti1a, and VAMP3/4 [41,48]. Antibodies against syntaxin 5, Ykt6, GS15, or GS28 inhibit the early endosome-to-Golgi transport of STxB/STx1B in a permeabilized cell assay [48]. Soluble cytosolic domains of syntaxin 6, syntaxin 16, or VAMP4 also block STxB/STx1B transport from early endosomes to the Golgi [41].

### 2.7. Other Regulators

Other factors that are involved in the early endosome-to-Golgi transport of STxB/STx1B include V-ATPase and protein kinases. Inhibition of V-ATPase using bafilomycin A or siRNA-mediated knockdown of its subunit ATP6V0A2 reduces the entry of STxB/STx1B into the Golgi [51]. Upon chemical inhibition or siRNA knockdown of PKCδ, there is a decrease in the overlap of STxB/STx1B with the Golgi marker giantin, and an increase with the early endosome marker EEA1 [50]. Similarly, inhibition or knockdown of p38 also causes a reduction in the overlap between STxB/STx1B and giantin [49].

## 3. Early Endosome-to-Golgi Transport of STx2

The molecular details of the retrograde trafficking of STx2 B-subunit (STx2B) only recently began to be elucidated [14,20,58]. STx2B traffics via the same retrograde pathway as STxB/STx1B and evades late endosomes/lysosomes by undergoing direct early endosome-to-Golgi trafficking as well (Figure 1A,B) [14]. Below, we describe similarities and differences in the mechanisms of trafficking of STx2B and STxB/STx1B, and we highlight results of a recent genome-wide siRNA screen that identified an unexpected role for late endosome-lysosome fusion in the early endosome-to-Golgi transport of STx2B [20,58].

### 3.1. Similarities and Differences in the Transport of STx2B and STxB/STx1B

**Figure 3:**
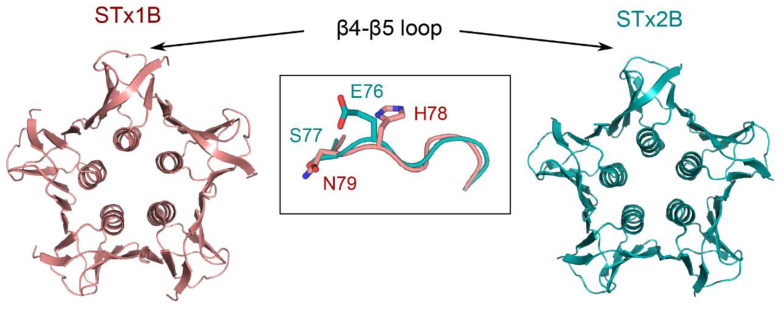
Crystal structures of STxB/STx1B and STx2B (Protein Data Bank (PDB): 1R4Q and 1R4P, respectively) with their β4–β5 loops.

Similar to STxB/STx1B, retrograde trafficking of STx2B requires dynamin, epsinR, retromer component Vps26, and the SNARE protein syntaxin 5 (Table 2) [14]. However, early endosome-to-Golgi traffic of STx2 has slower kinetics than that of STxB/STx1B, and STx2B takes a longer time to reach the Golgi than STxB/STx1B [14]. A striking difference between the toxins is that early endosome-to-Golgi transport of STx2B is GPP130-independent [10]. Unlike STxB/STx1B, STx2B does not bind GPP130 [10]. In STxB/STx1B, residues H78 and N79 in a surface-exposed loop (β4–β5 loop) are required for binding GPP130 (Figure 3). Alanine mutations at these sites abolish GPP130 binding and block the early endosome-to-Golgi transport of STx1B [10]. However, the corresponding residues in STx2B are E76 and S77 (Figure 3), respectively, explaining the lack of interaction between STx2B and GPP130 [10]. Nevertheless, the spatial orientation of the β4–β5 loop is nearly identical between STxB/STx1B and STx2B (Figure 3), and alanine mutations of E76 and S77 in STx2B also block the early endosome-to-Golgi transport of STx2B [14]. Hence, STxB/STx1B and STx2B use a conserved structural motif for early endosome-to-Golgi transport, raising the possibility that STx2B may also employ a yet unidentified endosomal receptor to traffic from early endosomes to the Golgi.

### 3.2. Late Endosome–Lysosome Fusion Modulates Early Endosome-to-Golgi Trafficking of STx2B—Insights Obtained from a Genome-Wide siRNA Screen

**Figure 4:**
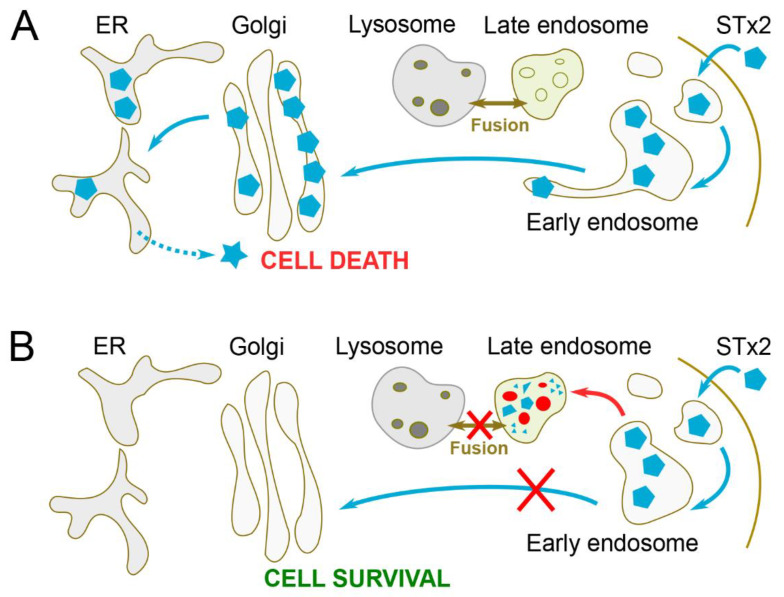
Role of late endosome–lysosome fusion in STx2 transport. (**A**). Under control conditions when late endosomes can undergo fusion with lysosomes, STx2B traffics from early endosomes to the Golgi. (**B**). When late endosome–lysosome fusion is inhibited, STx2B fails to exit early endosomes to traffic to the Golgi and, instead, is degraded in late endosomes. The underlying molecular mechanisms are unclear, but available evidence suggests that late endosome–lysosome fusion plays a critical role in the recruitment of retromer to early endosomes.

In an effort to comprehensively understand the commonalities and differences in the mechanisms of retrograde trafficking of STxB/STx1B and STx2B, as well as identify druggable host processes required for toxin trafficking, a viability-based genome-wide siRNA screen was performed in 2017 [58]. The primary screen identified 564, 535, and 196 genes (or hits) that, respectively, are required for toxicity induced by STx1-only, STx2-only, and both toxins [58]. Subsequent efforts focused on 35 hits required for STx2-only toxicity and known or predicted to generate proteins that localize to endosomes/Golgi, because these factors were most likely to specifically modulate the endosome-to-Golgi trafficking of STx2. A secondary screen revealed that 12 of these 35 hits reproducibly protect against STx2-toxicity when depleted (UNC50, B4GALT5, FUT1, KDR/VEGFR2, SNX14, STAM, TPCN1, SNX1, ASTN2, ATP11A, AP1AR, and Rab2a) (Table 2) [58]. Subsequent studies revealed that depletion of UNC50, Rab2a, STAM, or FUT1 blocks endosome-to-Golgi transport of STx2B and, notably, also impacts lysosomes and/or the autophagy pathway (Table 2) [20,58]. Three other factors in this list, TPCN1, SNX14, and KDR/VEGFR2, are also known modulators of lysosomal function and/or autophagy [59,60,61]. The above findings raised the interesting hypothesis that biogenesis or function of lysosomes and/or autophagy is essential for the trafficking and toxicity of STx2. This hypothesis was tested by leveraging several facts. Firstly, in the endolysosomal pathway, early endosomes mature into late endosomes that fuse with lysosomes, whereas, in the autophagic pathway, lysosomes fuse with autophagosomes [62,63,64,65,66]. Secondly, both the above membrane fusion events require the HOPS (homotypic fusion and vacuole protein sorting) tethering complex [62,64,65], but ATG7 is required for the formation of autophagosomes [67], and the SNARE syntaxin 17 is required for the fusion of autophagosomes, but not late endosomes, with lysosomes [62]. Studies revealed that inhibition of autophagosome formation, by depletion of ATG7, or autophagosome-lysosome fusion, by depletion of syntaxin 17, has no effect on the early endosome-to-Golgi trafficking of STx2B [20]. However, depletion of the HOPS complex protein Vps39, which inhibits fusion of lysosomes with late endosomes, as well as autophagosomes, robustly blocks the transport of STx2B from early endosomes to the Golgi, and reroutes STx2B to late endosomes for degradation (Figure 4A,B and Table 2) [20]. Put together, the above results imply that the early endosome-to-Golgi transport of STx2B depends on the fusion of late endosomes with lysosomes, but not on the autophagy pathway (Figure 4A,B). The mechanisms via which late endosome–lysosome fusion modulate early endosome-to-Golgi trafficking of STx2B are still being determined. Available preliminary evidence suggests that recruitment of retromer to early endosomal membranes is altered when late endosome–lysosome fusion is inhibited, providing a possible reason for the observed block in the early endosome-to-Golgi trafficking of STx2B [20].

## 4. Small-Molecule Inhibitors Targeting Early Endosome-to-Golgi Transport of STx, STx1, and STx2

Over the last few years, targeted efforts were made to generate inhibitors that block the trafficking of, and protect against toxicity induced by, STx/STx1 and STx2. These are summarized below.

### 4.1. Compound 134

**Figure 5:**
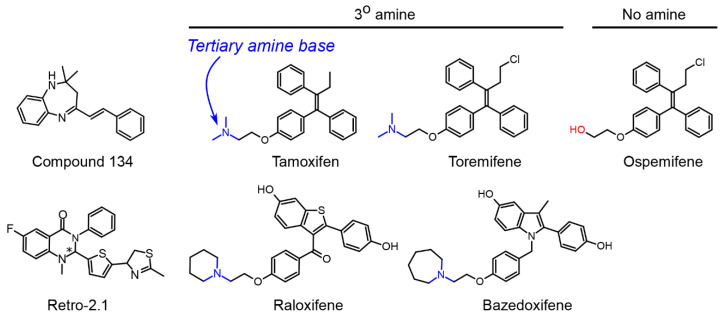
Small-molecule inhibitors of STx/STx1 and/or STx2 trafficking. Compound 134 (active concentration 50 µM) and the *S*-enantiomer of retro2.1 (median effective concentration (EC_50_) 54 nM) were tested only against STx/STx1, but the parent retro-2 compound is active against both STx/STx1 and STx2 at 20 µM. Tamoxifen is active against STx1 and STx2 at 2.5 to 10 µM. Tamoxifen derivatives with the tertiary amine base (tested at active concentrations of 10 µM) are protective against STx2, but were not tested against STx/STx1. Ospemifene lacks the tertiary amine base and is not protective.

Saenz et al. performed a high-throughput screen of 14,400 compounds with the goal of identifying compounds that protect against STx cytotoxicity in cells [17]. This screen identified one compound, #134 (Figure 5), which inhibits the endosome-to-Golgi transport of STxB/STx1B and protects against STx toxicity when used at a concentration of 50 µM in Vero cells [17]. A second compound, #75, which inhibits toxin transport to perinuclear endosomes at a concentration of 25 µM was also identified [17]. These compounds were primarily developed as tools to study retrograde trafficking, and their therapeutic potential was not explored. Indeed, both compounds impact Golgi morphology [17], which may limit their translational potential.

Of note, the above screen subsequently led to the identification of a third compound, named Golgicide A, which also blocks the endosome-to-Golgi transport of STxB/STx1B [68]. However, Golgicide A is an inhibitor of the ARF guanine exchange factor GBF1 and has pleiotropic effects on membrane trafficking [68], which makes it unsuitable for clinical development.

### 4.2. Retro-2 Substances

Retro compounds emerged from a high-throughput screen for small molecules that protect cells from ricin and STx toxins in 2010 (Figure 5) [18]. Two identified compounds, retro-1 and retro-2, provide 10 to 100-fold protection against STx1 and STx2 in HeLa cells, and block the early endosome-to-Golgi transport of STxB/STx1B when used at a concentration of 20 µM [18]. Initial studies revealed that both retro-1 and retro-2 relocalize the SNARE protein syntaxin 5 from its normal site of accumulation on perinuclear Golgi membranes [18]. A more detailed mechanism was recently provided with the discovery that retro-2 targets the endoplasmic reticulum exit site component Sec16A and affects the anterograde transport of syntaxin 5 from the endoplasmic reticulum to the Golgi [27]. An alternative mechanism was also proposed whereby retro-2 enhances syntaxin 5 degradation and alters its subcellular localization by blocking its delivery to the endoplasmic reticulum targeting factor TRC40, which is required for the insertion of syntaxin 5 into the endoplasmic reticulum [69]. Structure–activity studies to improve the potency of retro analogues were performed, and the best in class molecule is *(S*) retro-2.1 (Figure 5), which has an median effective concentration (EC_50_) of 54 nM against STx in HeLa cells [70,71]. In animal studies, retro-2 protects mice against toxicity induced by the plant toxin ricin, which also traffics into cells via the retrograde pathway [18]. Furthermore, a cyclized form of retro-2, termed retro-2^cycl^, reduces morbidity and mortality in mice infected with an STEC O104: H4 that produces STx2 when used at a dose of 100 mg/kg [72].

Retro compounds are reported to have a selective inhibitory effect on the trafficking of exogenous cargo like STx, but lack effects on compartment morphology, endogenous retrograde cargos, or other intracellular trafficking pathways [18,27]. This is surprising given the ubiquitous role of syntaxin 5 in membrane trafficking. The effect of retro compounds on syntaxin 5 (and now Sec16a) raises the possibility of pleotropic effects on membrane trafficking in vivo, and it highlights the need for good lab practice animal toxicology studies to determine their safety profile, before phase I trials in humans can be contemplated.

### 4.3. Manganese

A serendipitous discovery in the field was that exposure to 0.5–500 µM manganese induces rapid degradation of GPP130, the endosomal receptor of STxB/STx1B (Figure 2B) [73,74,75]. Initial studies revealed that the mechanism related to an increase in intra-Golgi manganese, which reroutes GPP130 to late endosomes/lysosomes from the Golgi apparatus for degradation (Figure 2B) [73,74]. Subsequently, it was discovered that the increased intra-Golgi manganese levels induce GPP130 oligomerization, which acts as a trafficking signal to reroute the protein to the degradative pathway [76]. This line of work led to the important discovery of a novel quality control mechanism in the Golgi through which aggregated proteins, in general, are targeted to lysosomes for degradation [76,77]. More relevant to the current discussion, as GPP130 is required for STxB/STx1B transport (Figure 2A), the protective effect of manganese against STx1 was then tested. Mechanistic assays in cell culture revealed that manganese blocks the early endosome-to-Golgi transport of STxB/STx1B; under these conditions, STxB/STx1B is rerouted to late endosomes/lysosomes and degraded (Figure 2B) [19]. This effect is rescued by expression of manganese-insensitive versions of GPP130 [19]. Moreover, in cells, treatment with 125–500 µM manganese provided a protection factor of 3800 against STx1-induced cell death [19]. Importantly, in mice, treatment with 10–50 mg manganese/kg conferred complete protection against STx1-induced lethality and kidney damage without inducing overt manganese toxicity [19]. However, since the early endosome-to-Golgi transport of STx2B is GPP130-independent, manganese does not protect cells against STx2 under the same conditions [10]. Further development assays (safety, non-human primate, and clinical studies) are necessary before it can be determined whether manganese may be a safe therapeutic option to treat human patients infected with STx-producing bacteria (e.g., *Shigella*). Particular concerns are off-target effects because GPP130 is required for the endosome-to-Golgi trafficking of host proteins, such as GP73 and TGN46 [26], as well as the possibility that Mn therapy may induce neurotoxicity, despite the likely short duration necessary to counter STx-induced disease [78]. Moreover, due to the lack of effect against STx2, manganese by itself is unlikely to be effective against STEC.

### 4.4. Tamoxifen and Derivatives

Small molecules that increase the pH of the endolysosomal compartment inhibit the fusion of late endosomes with lysosomes [79,80]. Several drugs that are currently approved by the Food and Drug Administration (FDA) for the treatment of other diseases alter endolysosomal pH. As late endosome–lysosome fusion is necessary for early endosome-to-Golgi trafficking of STx2B (Figure 4A,B), Selyunin et al. hypothesized that FDA-approved drugs that affect endolysosomal pH may inhibit the trafficking and toxicity of STx2 [20]. A screen of such FDA-approved drugs identified the breast cancer chemotherapeutic tamoxifen to be a potent inhibitor of STx2 trafficking in HeLa cells (Figure 5) [20]. In tamoxifen-treated cells, trafficking of STx2B is inhibited at the early endosome-to-Golgi step, and the toxin is rerouted to late endosomes and degraded [20]. Treatment with 10 µM tamoxifen provides >100-fold protection against STx2-induced cell death, and the protective effect is evident at lower doses as well (2.5 or 5 µM) [20]. Similar levels of tamoxifen also block the trafficking of STx1 and protect cells against STx1-induced death [20]. Notably, in mouse experiments, treatment with 70 mg tamoxifen/kg, which corresponds to currently approved human dosing regimens, significantly improves survival after exposure to a lethal amount of STx1 or STx2 [20].

Tamoxifen is a selective estrogen receptor modulator that antagonizes the effects of estrogen and is, therefore, used for breast cancer therapy [81]. However, as described in Selyunin et al. [20], a less appreciated feature of tamoxifen is that it is a lysosomotropic weak base that accumulates within acidic endolysosomes and directly increases compartment pH [82,83]. The capability of tamoxifen to change endolysosomal pH is independent of estrogen receptors and, instead, depends on a tertiary amine in its structure that makes it a weak base (Figure 5) [82,83]. Based on this, Selyunin et al. hypothesized that activity of tamoxifen against STx1/STx2 depended on its tertiary amine base, and not estrogen signaling [20]. Notably, the cell-based assays described above [20] were performed in HeLa cells, which do not express estrogen receptors [84], indicating that the protective effect of tamoxifen against STx2 is independent of estrogen receptor modulation [20]. Furthermore, three clinically approved tamoxifen derivatives with the tertiary amine base, toremifene, raloxifene, and bazedoxifene, provide as much protection as tamoxifen against STx2, but ospemifene, which does not have an amine group and is not a weak base, fails to protect all together (all compounds used at 10 µM) (Figure 5) [20]. The results, in totality, imply that tamoxifen exerts its protective effect against STx1/STx2 by acting as a weak base that directly increases endolysosomal pH, and not by acting as a selective estrogen receptor modulator [20]. As tamoxifen, toremifene, raloxifene, and bazedoxifene are already approved for human use, it may be feasible to translate their protective activity against STx1/STx2 in experimental systems to human therapy more rapidly than other drugs in development [20].

## 5. Conclusions

Substantial details are available about the mechanisms of trafficking of STx/STx1 because these toxins are widely employed as a model retrograde cargo. Indeed, work on STx/STx1 was a major contributor to our current understanding of endosome-to-Golgi trafficking. More recent studies on STx2, however, highlight the importance of cargo-specific differences and the fact that results obtained from STx/STx1 may not necessarily be applicable to related toxins. Critical issues that remain to be addressed include understanding how STxB/STx1B along with its endosomal receptor GPP130 sorts into Golgi-directed endosomal tubules, testing whether STx2B utilizes an endosomal receptor for exiting early endosomes, elucidating the mechanisms by which two structurally similar toxins, STx1 and STx2, utilize different molecular factors to undergo retrograde transport, and determining whether the principles of trafficking utilized by STx/STx1 and STx2 are more generally applicable to all AB_5_ toxins. Additionally, most trafficking studies are performed in model cell lines (e.g., HeLa or Vero), and applicability of results to disease-relevant systems (renal cell lines, organoids, and animal models) is another important area of future work. Basic science studies on STx/STx1 and STx2 trafficking already led to the development of experimental drugs that inhibit toxin transport, underlining the translational and human health potential of this line of research.

## Figures and Tables

**Table 1:** Factors required for early endosome-to-Golgi transport of STxB/STx1B.

Factor	Function	Reference
GPP130	Endosomal sorting receptor	[19]
Cholesterol	Microdomain organization	[28]
Clathrin	Coat	[30,31]
EpsinR	Clathrin adaptor	[30]
OCRL1	Phosphatidylinositol 4,5-bisphosphate 5-phosphatase	[32]
Retromer (SNX1, SNX2, Vps26/29/35)	Coat	[33,34,35]
EHD3	Retromer interactor	[36]
Rabenosyn5	EHD3 interactor	[36]
RME-8	Recruit Hsc-70 to SNX1	[37]
Hsc-70	Clathrin uncoating ATPase	[37]
Hrs	Clathrin interactor	[37]
Dynamin	Membrane scission	[31]
Microtubule	Cytoskeleton	[38]
Dynein	Motor	[38]
Actin	Cytoskeleton	[39]
Cdc42	Rho GTPase	[39]
ARHGAP21	Cdc42 activating protein	[39]
Rab11	Small GTPase	[40,41]
Rab6a’	Small GTPase	[41,42]
Arl1	Small GTPase, Golgin anchor	[43]
Golgin 97	Tether, Arl1 effector	[43]
Golgin 245	Tether, Arl1 effector	[44]
GCC185	Tether, Arl1 effector	[45]
TMF	Tether, Rab6 effector	[46]
GARP	Tether	[47]
Syntaxin 5, Ykt6, GS15, GS28	SNARE complex	[48]
Syntaxin 6, syntaxin 16, Vti1a, Vamp3/4	SNARE complex	[41]
p38	Kinase	[49]
PKCδ	Kinase	[50]
V-ATPase	Proton pump	[51]

**Table 2:** Factors required for the endosome-to-Golgi transport of STx2B.

Factor	Function	Reference
Dynamin	Membrane scission	[14]
EpsinR	Clathrin adaptor	[14]
Vps26	Retromer component	[14]
Syntaxin 5	SNARE complex component	[14]
UNC50	GBF1 interactor, recruits GBF1 to the Golgi	[58]
Rab2a	Small GTPase	[20]
STAM	ESCRT (endosomal sorting complexes required for transport)-0 complex component	[20]
FUT1	Fucosylation enzyme	[20]
Vps39	HOPS (homotypic fusion and vacuole protein sorting) complex component	[20]
